# Expanded Glucose Import Capability Affords *Staphylococcus aureus* Optimized Glycolytic Flux during Infection

**DOI:** 10.1128/mBio.00296-16

**Published:** 2016-06-21

**Authors:** Nicholas P. Vitko, Melinda R. Grosser, Dal Khatri, Thurlow R. Lance, Anthony R. Richardson

**Affiliations:** Department of Microbiology and Immunology, University of North Carolina at Chapel Hill, Chapel Hill, North Carolina, USA

## Abstract

Acquisition of numerous virulence determinants affords *Staphylococcus aureus* greater pathogenicity than other skin-colonizing staphylococci in humans. Additionally, the metabolic adaptation of *S. aureus* to nonrespiratory conditions encountered during infection (e.g., hypoxia, nitric oxide, iron chelation) has been implicated as contributing to *S. aureus* virulence. Specifically, *S. aureus* has been shown to ferment glycolytic substrates in nonrespiratory environments encountered within the host. Here, we show that *S. aureus* has acquired unique carbohydrate transporters that facilitate the maximal uptake of host sugars and serve to support nonrespiratory growth in inflamed tissue. The carbohydrate substrates of 11 *S. aureus* transporters were identified, and at least four of their genes encode *S. aureus* glucose transporters (*glcA*, *glcB*, *glcC*, and *glcU*). Moreover, two transporter genes (*glcA* and *glcC*) are unique to *S. aureus* and contribute disproportionately to the nonrespiratory growth of *S. aureus* on glucose. Targeted inactivation of sugar transporters reduced glucose uptake and attenuated *S. aureus* in a murine model of skin and soft tissue infections. These data expand the evidence for metabolic adaptation of *S. aureus* to invasive infection and demonstrate the specific requirement for the fermentation of glucose over all other available carbohydrates. Ultimately, acquisition of foreign genes allows *S. aureus* to adopt a metabolic strategy resembling that of infiltrating host immune cells: high glycolytic flux coupled to lactate excretion.

## IMPORTANCE

The bacterial pathogen *Staphylococcus aureus* causes a wide range of human infections that are costly and difficult to treat. *S. aureus* differs from closely related commensal staphylococci in its ability to flourish following the invasion of deeper tissue from the skin surface. There, *S. aureus* primarily uses glucose to grow under respiration-limiting conditions imposed by the immune system. It was previously unclear how *S. aureus* thrives in this environment when other *Staphylococcus* species cannot. Our results provide evidence that *S. aureus* has acquired an expanded repertoire of carbohydrate transporters. In particular, four glucose transporters contribute to efficient *S. aureus* growth during infection. Thus, *S. aureus* has evolved to maximize its glucose uptake abilities for enhanced glycolytic flux during tissue invasion. This dependence on glucose acquisition for *S. aureus* virulence may also explain links between serious infectious complications associated with diabetic patients exhibiting elevated blood glucose levels.

## INTRODUCTION

*Staphylococcus aureus* is a Gram-positive coccus that asymptomatically colonizes healthy human skin (1, 2). However, a compromised skin barrier or mucous membrane can lead to severe *S. aureus* infections, including: skin and soft tissue infections (SSTIs), bacteremia, osteomyelitis, pneumonia, and toxic shock syndrome (3–5). Many other species of staphylococci (e.g., *S. epidermidis*, *S. haemolyticus*, *S. saprophyticus*, etc.) also colonize human skin but cause disease far less frequently and with less severity than *S. aureus* (6). This difference has been extensively studied and is generally attributed to the combined presence of numerous unique virulence factors in the *S. aureus* genome, such as toxins, adhesins, antiphagocytic factors, and protein A (7–9). Absent from this explanation is the contribution of metabolic adaptation.

The *S. aureus* life cycle can plausibly be described as low-level growth on the skin surface with periodic penetration of deeper tissue environments marking a phase of enhanced growth and increased incidence of transmission. Major physiological differences between the skin surface and underlying tissue include oxygen concentrations, micronutrient availability, nitrogen sources, carbon sources, and pH (10–12). In general, the skin surface has lower levels of carbohydrates and peptides, relatively high levels of oxygen, and an acidic pH. Sterile tissue, on the other hand, contains an abundance of carbohydrates and peptides, lower levels of free oxygen, and a more neutral pH. However, invasion of sterile tissue by *S. aureus* leads to the activation of several innate immune responses that combine to limit bacterial respiration (e.g., iron chelation, nitric oxide [NO] production, and robust oxygen consumption by innate immune cells) (13–17). Thus, natural selection would dictate that *S. aureus* has adapted to take advantage of the unique metabolites present within sterile tissue (e.g., peptides and carbohydrates) in a manner compatible with increased resistance to host inflammation (i.e., respiration inhibition).

Recently, we demonstrated that *S. aureus* requires both glycolysis and lactate fermentation for SSTIs and bloodstream infections and that only carbohydrates support the growth of *S. aureus* under both high NO stress and anaerobiosis (i.e., nonrespiratory conditions) (13, 16). Additionally, the lack of abundant iron during infection limits respiration and necessitates high glycolytic flux coupled to lactate excretion (15, 17). This metabolic strategy, which is similar to that of activated immune cells, allows for the generation of ATP in a redox-balanced, respiration-independent manner. However, aside from the presence of a unique lactate dehydrogenase gene (*ldh1*) in the *S. aureus* genome that promotes enhanced redox balancing during respiration inhibition, there is a lack of molecular evidence supporting a contribution of metabolic adaptation to infection as a distinguishing characteristic of *S. aureus* (13). Given the high evolutionary conservation of glycolysis among the kingdoms of life, we postulated that metabolic adaptation to promote high glycolytic flux would most easily be achieved by the acquisition of additional carbohydrate importers (18).

Bacterial carbohydrate transporters can be divided into those that modify the sugar during transport (i.e., phosphotransferase system [PTS] transporters) and those that do not (i.e., primary and secondary active transporters) (19–21). PTS transport proceeds via a phosphorelay system that transfers the phosphoryl group of phosphoenolpyruvate (PEP) through a series of carrier proteins (EI and HPr) to a transporter (EII) and then on to the sugar as it is imported. PTS sugar transporters are composed of at least three subunits: EIIA, EIIB, and EIIC. The EIIA and EIIB subunits transfer the phosphoryl group from HPr to the sugar, while the EIIC subunit acts as a sugar-specific transmembrane receptor. Interestingly, the EII subunits may be encoded as individual polypeptides or fused into multisubunit proteins. PTS-dependent carbohydrate transport is unique to bacteria and is the predominant form of sugar uptake. PTS-dependent transport is also functionally linked to the transcriptional regulation of cellular metabolism via carbon catabolite repression (mediated by CcpA in Gram-positive bacteria), which further contributes to overall metabolic efficiency.

In this report, we show that *S. aureus* exhibits better nonrespiratory growth than other skin-dwelling staphylococci and partially attribute this phenomenon to an increased capacity for carbohydrate import. More specifically, we identify the carbohydrate substrates for 11 putative sugar transporters and demonstrate that *S. aureus* exhibits preferential uptake of glucose during infection as a result of the combined activities of at least four glucose transporters, two of which are newly acquired and therefore unique to *S. aureus*.

## RESULTS

### *S. aureus* exhibits better anaerobic growth than other staphylococci.

**FIG 1:**
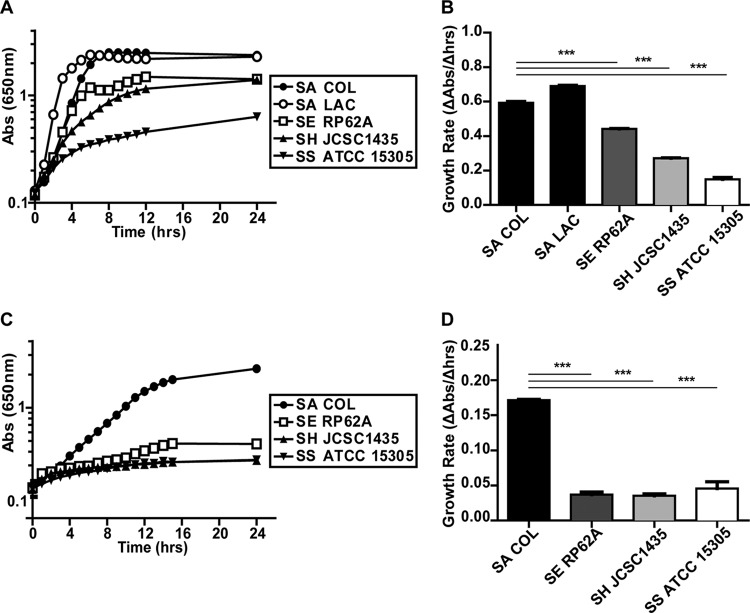
*S. aureus* displays better anaerobic growth than CoNS. Anaerobic growth of *S. aureus* (SA) COL and LAC, *S. epidermidis* (SE) RP62A, *S. haemolyticus* (SH) ATCC 29970, and *S. saprophyticus* (SS) ATCC 15305 in TSB (A) and CDM plus 25 mM glucose (C) (*n* = 3). Corresponding average growth rates for TSB and CDM plus glucose are displayed in panels B and D, respectively (*n =* 3; error bars show the pooled standard error of the mean). Growth rates were calculated from 2 to 4 h (*S. aureus* LAC) and 3 to 5 h (*S. aureus* COL, *S. epidermidis*, *S. haemolyticus*, and *S. saprophyticus*) in TSB and from 2 to 8 h in CDM plus 25 mM glucose. Statistical significance was calculated with a Student two-sided *t* test (***, *P* ≤ 0.001). Abs, absorbance.

*S. aureus* grows in the presence of NO levels that inhibit both respiration and the growth of other staphylococci (13). To test whether this enhanced growth behavior occurs under other nonrespiratory conditions, we compared the anaerobic growth of *S. aureus* strains COL and LAC to that of *S. epidermidis* RP62A, *S. haemolyticus* ATCC 29970, and *S. saprophyticus* ATCC 15305 in a rich medium. Both strains of *S. aureus* exhibited better growth than the other *Staphylococcus* species, as evidenced by significantly greater growth rates and terminal optical densities (ODs) (Fig. 1A and B). Next, we compared the anaerobic growth of *S. aureus* COL to that of the other staphylococcal species in chemically defined medium (CDM) with glucose as the primary carbon source. Once again, *S. aureus* exhibited better anaerobic growth than the other *Staphylococcus* species (Fig. 1C and D). These data suggest that unique glycolytic and/or fermentative capabilities account for the enhanced growth of *S. aureus* under nonrespiratory conditions.

### *S. aureus* encodes an expanded repertoire of predicted carbohydrate transporters.

**FIG 2:**
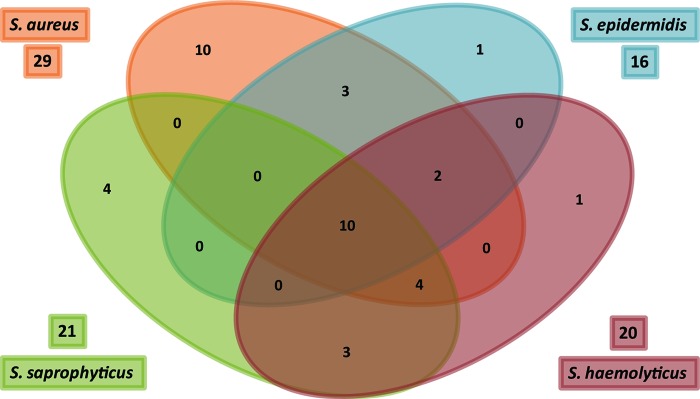
*S. aureus* Encodes enhanced carbohydrate transport capability. Shown is a Venn diagram depicting the presence and conservation of putative carbohydrate transport proteins in the genomes of *S. aureus* COL, *S. epidermidis* RP62A, *S. haemolyticus* JCSC1435, and *S. saprophyticus* ATCC 15305. *S. aureus* encodes more overall transporters (*n* = 22) and the highest number of unique transporters not found in any of the other species (*n* = 10).

One explanation for the increased growth rate of *S. aureus* under nonrespiratory conditions could be an increased capacity to import fermentable carbohydrates. To test this hypothesis, we performed a comparative genome analysis of putative carbohydrate transporters encoded by *S. aureus* COL, *S. aureus* LAC, *S. epidermidis* RP62A, *S. haemolyticus* JCSC1435, and *S. saprophyticus* ATCC 15305. We found that *S. aureus* encodes the largest total number of carbohydrate transporters (22), as well as the most unique carbohydrate transporters (10) (Fig. 2; see Table S1 in the supplemental material). Interestingly, 4 of the 10 unique *S. aureus* alleles are predicted to encode glucose transporters: SAUSA300_0191 (*glcA*), SAUSA300_0194, SAUSA300_0236 (*glcC*), and SAUSA300_0259 (see Table S1). Importantly, glucose is (i) largely absent from the skin surface, (ii) the most abundant free carbohydrate in human serum, and (iii) used by activated innate immune cells to both produce and resist inflammatory radicals.

### Carbohydrate uptake in *S. aureus* is mostly PTS dependent and contributes disproportionately to nonrespiratory growth.

The majority (21/29) of putative *S. aureus* carbohydrate transport proteins are predicted to be PTS proteins. Thus, we decided to test the contribution of PTS-dependent carbohydrate transport to the nonrespiratory growth of *S. aureus* by using a PTS-deficient stain of *S. aureus* (*ptsH-*H15A). The H15A substitution in PtsH prevents the transfer of the phosphoryl group from EI to PtsH, thereby inhibiting PTS-dependent sugar uptake but not directly affecting interactions with CcpA, the master regulator of carbon catabolite repression (21). To confirm the efficacy of this mutation and identify PTS-dependent substrates, we compared the aerobic growth of wild-type (WT) and *ptsH*-H15A mutant *S. aureus* on 16 different carbohydrates. Previous studies demonstrated PTS-dependent utilization of at least seven carbohydrates (lactose, fructose, galactose, maltose, sucrose, glucose, and mannitol) by *S. aureus* (23). In line with these observations, we found that loss of PTS-dependent sugar uptake prevented *S. aureus* growth on 10 carbohydrates (mannose, fructose, galactose, mannitol, *N*-acetylglucosamine, *N*-acetylmannosamine, maltose, sucrose, trehalose, lactose, and turanose) and reduced its growth on two carbohydrates (glucose and maltotriose) but did not affect its growth on ribose (Table 1). These data show that *S. aureus* carbohydrate utilization is largely PTS dependent.

**TABLE 1:** Identification of PTS-dependent carbohydrates that support growth of *S. aureus*

Sugar	Growth of:			
	COL		LAC	
	WT	*ptsH*-H15A mutant	WT	*ptsH*-H15A mutant
Glucose	+++^a^	++	+++	++
Mannose	+++		+++	
Fructose	+		+++	
Galactose	+		+	
Ascorbate				
Mannitol	+++		+++	
Sorbitol				
GlcNAc	++		++	
ManNAc	+		+	
Ribose	++	++	+	++
Maltose	+++		+++	
Sucrose	+++		+++	
Trehalose	++		++	
Lactose	++		++	
Turanose	+		+	
Maltotriose	+++	+	+++	++

a +++, grows as well as when cultured with glucose; ++, grows to same terminal OD as when cultured on glucose, but delayed ≥10 h; +, does not grow to maximal terminal OD.

**FIG 3:**
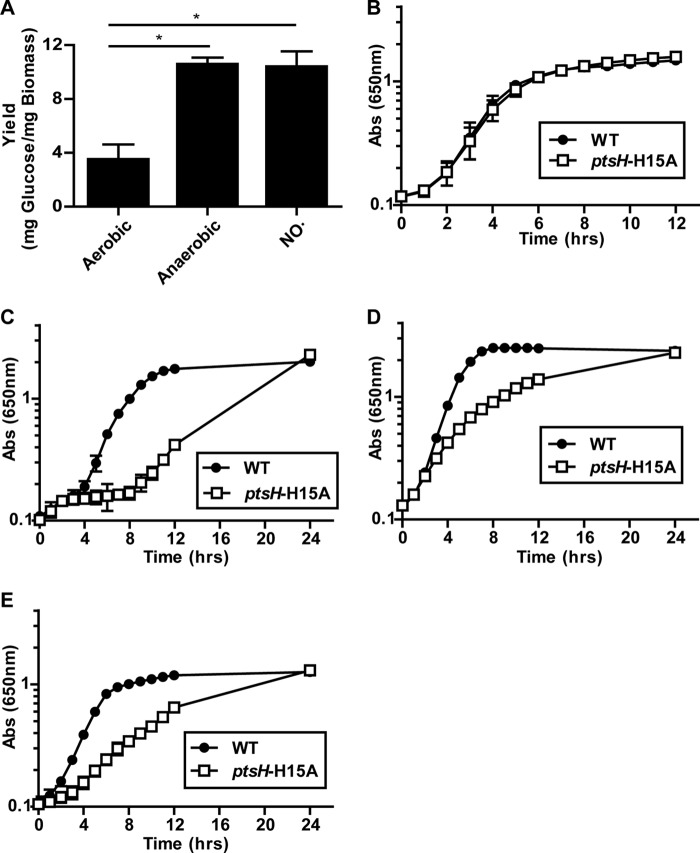
PTS-dependent carbohydrate uptake contributes disproportionately to the nonrespiratory growth of *S. aureus*. (A) Glucose yield (milligrams of glucose consumed per milligram [dry weight] of biomass) of *S. aureus* COL under respiratory and nonrespiratory (anaerobic and NO-stressed) conditions (*n =* 3; error bars show the standard error of the mean). *S. aureus* consumes ~3-fold more glucose per cell under nonrespiratory conditions. Statistical significance was calculated with a Student two-sided *t* test (*, *P* ≤ 0.01). (B to E) Growth curves of WT and PTS-deficient (*ptsH*-H15A) *S. aureus* LAC in TSB under aerobic (B), anaerobic (C), NO-stressed (D), and metal-limiting (E) conditions (*n =* 3). Abs, absorbance.

Nonrespiratory fermentation of carbohydrates is inherently less energy efficient than respiration. Consequently, *S. aureus* must consume three times the amount of glucose under fermentative growth as under respiratory conditions in order to produce equivalent biomass (dry weight) (Fig. 3A). Maintaining this elevated level of sugar catabolism necessitates more efficient uptake of carbohydrates, particularly under nonrespiratory conditions. Accordingly, despite the large reduction in carbohydrate utilization in the PTS-deficient strain, aerobic growth of *S. aureus* LAC in complex medium is largely unaffected (Fig. 3B). In contrast, the PTS-deficient mutant exhibited a significant reduction in growth under anaerobic conditions (Fig. 3C), under NO stress (Fig. 3D), and under metal-limited conditions (Fig. 3E). Similar results were obtained with *S. aureus* COL (data not shown). Thus, the increased reliance of *S. aureus* on carbohydrate transport during nonrespiratory growth implicates carbohydrate transporter acquisition as a possible mechanism of metabolic adaption of *S. aureus* to infection.

### Substrate identification for individual *S. aureus* PTS proteins.

To identify the sugar specificity of individual *S. aureus* PTS proteins, we screened mutants with insertions in all of the predicted PTS protein-encoding genes for aerobic growth defects on selected carbohydrates. Previous studies identified *fruA* and *mtlFA* as encoding fructose and mannitol importers, respectively (24, 25). In support of these observations, we found that the *mtlF* and *mtlA* mutants were unable to grow on mannitol, while *S. aureus* COL, a natural *fruA* mutant, exhibited poorer growth than *S. aureus* JE2 on fructose. Additionally, we identified PTS transport proteins contributing to the uptake of nine other carbohydrates. Overall, our results link individual PTS transport proteins to the uptake of 11 of the 12 PTS-dependent sugars (Table 2). All phenotypes were confirmed in a second *S. aureus* strain (COL) background following transduction (data not shown). Consistent with the growth phenotypes of the *ptsH*-deficient mutant, not a single *S. aureus* PTS transposon mutant displayed a growth defect on glucose, suggesting that glucose uptake (i) is genetically redundant and (ii) likely requires both PTS and non-PTS-dependent transporters.

**TABLE 2:** Individual PTS Tn insertions tested for growth on a subset of utilizable carbon sources

SAUSA300 Tn insertion locus	Gene name	EII subunit(s)	Family	Phenotype on:											
				Glucose	Mannose	Fructose	Galactose	Mannitol	GlcNAc	ManNAc	Maltose	Sucrose	Trehalose	Lactose	Maltotriose
None															
0191	*ptsG*/*glcA*	ABC	PTS-Glc		PL^a^				CL^a^						
0194		BC	PTS-Glc												
0236		BC	PTS-Glc							CL^a^					
0239		A	PTS-Gat												
0240		B	PTS-Gat												
0241		C	PTS-Gat												
0259		A	PTS-Glc												
0332		C	PTS-Asc												
0448		BC	PTS-Glc										CL^a^		PL^a^
1315		ABC	PTS-Fru												PL^a^
1672		BC	PTS-Glc												
2105	*mtlA*	BC	PTS-Fru					CL^a^							
2107	*mtlF*	A	PTS-Fru					CL^a^							
2150	*lacE*	BC	PTS-Lac				CL^a^							CL^a^	
2151	*lacF*	A	PTS-Lac				CL							CL	
2270		BC	PTS-Glc								PL^a^				PL^a^
2324		BC	PTS-Glc									PL^a^			
2476	*glcB*	ABC	PTS-Glc												
2576		ABC	PTS-Fru		PL										

a Phenotype confirmed with an *S. aureus* COL transductant: CL, complete loss of growth; PL, partial loss of growth.

### *S. aureus* glucose transport is highly redundant.

**FIG 4:**
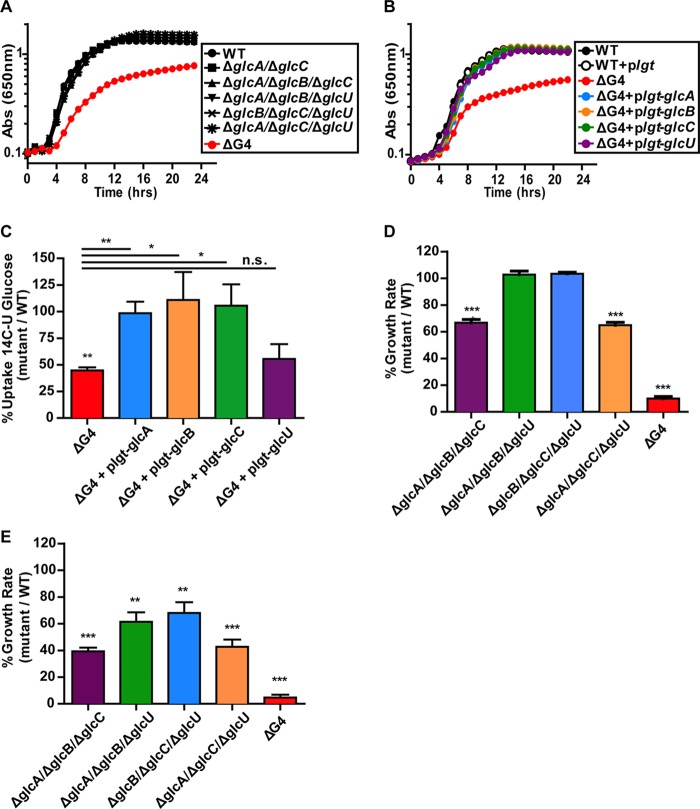
Contributions of the identified glucose transporters to the nonrespiratory growth of *S. aureus*. (A) Aerobic growth of WT and selected double, triple, and quadruple *S. aureus* COL glucose transporter mutants in CDM plus 25 mM glucose (*n* = 3). (B) Representative aerobic growth curve demonstrating complementation of *S. aureus* COL ΔG4 mutant growth in CDM plus 25 mM glucose by each individual glucose transporter (*n* = 3). (C) Percent [U-^14^C]glucose uptake by *S. aureus* COL ΔG4 relative to that of the WT, as well as ΔG4 complemented with each individual glucose transporter gene. Uptake by each strain was measured following 12 min of incubation with radiolabeled substrate and then normalized to that of the WT (*n =* 4; error bars show the standard error of the mean). Statistical significance was calculated with a Student two-sided *t* test (*, *P* ≤ 0.05; **, *P* ≤ 0.01; ***, *P* ≤ 0.001). (D and E) Nonrespiratory growth rate of *S. aureus* COL ΔG4, relative to that of the WT, compared to that of mutants expressing individual transporter genes from their native promoters. Strains were cultured anaerobically (D) or under NO stress (10 mM NOC-12–1 mM DEA-NO) (E) (*n =* 3; error bars show the pooled standard error of the mean). Statistical significance was calculated with a Student two-sided *t* test (**, *P* ≤ 0.01; ***, *P* ≤ 0.001). Abs, absorbance.

To identify the *S. aureus* glucose transporters, four different candidate genes were mutated via allelic replacement (three PTS transporters [*glcA*, *glcB*, and SAUSA300_0236] and one non-PTS transporter [*glcU*]) and then combined into all possible double, triple, and quadruple mutants. The deleted genes were chosen on the basis of a combination of sequence similarity to known glucose transporters and high expression levels during aerobic growth on glucose (data not shown). We found that only the *S. aureus* quadruple mutant (Δ*glcA* Δ*glcB* Δ*glcU* ΔSAUSA300_0236) exhibited a substantial aerobic growth defect on glucose (the quadruple mutant is referred to here as *S. aureus* ΔG4, and the SAUSA300_0236 gene is referred to as *glcC*) (Fig. 4A). Additionally, each putative glucose transporter was able to independently complement the aerobic growth defect of the *S. aureus* ΔG4 mutant on glucose (Fig. 4B). Lastly, we found that attenuation of ΔG4 mutant growth was fairly specific to glucose (see Table S2 in the supplemental material) (ManNAc and GlcNAc were not tested, as GlcA and GlcC were previously implicated in their transport; see Table S1 in the supplemental material).

To confirm that *glcA*, *glcB*, *glcC*, and *glcU* encode glucose transporters, we performed radiolabeled-glucose uptake assays with WT *S. aureus*, the ΔG4 mutant, and the ΔG4 mutant containing plasmids constitutively expressing each of the four glucose transporters. The ΔG4 mutant exhibited significantly less glucose uptake than WT *S. aureus* (45% of the WT level) (Fig. 4C). Complementation of the ΔG4 mutant with *glcA*, *glcB*, *glcC*, or *glcU* increased its glucose uptake to 98, 110, 105, or 56% of the WT level, respectively. The lack of significant uptake complementation by *glcU* could be explained by the fact that *glcU* encodes a member of the glucose/ribose porter family, a family of secondary active transporters that rely on proton motive force (PMF) for energy (26, 27). The dense cell pellet conditions required to perform these uptake assays likely have a negative impact on PMF. This would specifically decrease the activity of PMF-dependent GlcU more than that of the PEP-dependent PTS transporters GlcA, GlcB, and GlcC. Importantly, the *S. aureus* ΔG4 mutant is still capable of importing glucose and exhibits residual aerobic growth on glucose. These data indicate the presence of an additional glucose transporter(s).

To rule out the contribution of other PTS-dependent transporters to *S. aureus* glucose uptake, we compared the aerobic growth of WT and ΔG4, *ptsH*-H15A Δ*glcU*, and *ptsH*-H15A Δ*glk* mutant *S. aureus* COL. Glucose kinase, encoded by *glk*, is responsible for phosphorylating intracellular glucose taken up by non-PTS transporters. Thus, without *glk*, intracellular glucose cannot be catabolized unless transported via PTS proteins. The growth of the *S. aureus* ΔG4 and *ptsH*-H15A Δ*glcU* mutants was indistinguishable, whereas the *S. aureus* ptsH-H15A Δ*glk* mutant exhibited no residual growth in CDM with glucose as the primary carbon source (see Fig. S1 in the supplemental material). These data indicate that one or several unidentified non-PTS-dependent transporters are responsible for the remaining *S. aureus* ΔG4 glucose uptake observed.

### GlcA and GlcC contribute disproportionately to the nonrespiratory growth of *S. aureus* on glucose.

Next, we compared the aerobic, anaerobic, and NO-exposed growth of the various *S. aureus* glucose transporter mutants in CDM with glucose as the primary carbon source. The two triple mutants lacking both unique glucose transporters (GlcA and GlcC), and thus only expressing GlcB or GlcU, grew significantly more poorly than the other triple mutants, in which either GlcA or GlcC remained functional (Fig. 4D and E). This suggests that the unique glucose transporters GlcA and GlcC contribute disproportionately to *S. aureus* growth on glucose under nonrespiratory conditions. The ability of either GlcA or GlcC alone to individually maintain WT growth under nonrespiratory conditions cannot be explained by its expression level. The *glcA* transcript levels were commensurate with those of *glcB* and *glcU* (see Fig. S2 in the supplemental material). Moreover, *glcC* transcription was less robust under all of the conditions tested. Furthermore, none of the glucose transporter genes responded to the presence or absence of glucose or to respiratory inhibition, with the exception of *glcC*, which showed modest induction under anaerobiosis. Thus, other factors must explain the ability of GlcA or GlcC to fully restore growth by itself, such as translation efficiency, protein stability, and/or affinity for glucose.

### Rich medium provides alternative carbohydrates to support nonrespiratory growth of *S. aureus*.

We hypothesized that the acquisition of additional glucose transporters might partially explain the enhanced nonrespiratory growth phenotypes exhibited by *S. aureus* in both CDM and rich medium. Thus, we compared the growth of WT *S. aureus* (normal transport) with that of the ΔG4 (significantly reduced glucose uptake) and *ptsH*-H15A Δ*glcU* (severe defect in all carbohydrate import) *S. aureus* (COL and LAC) mutants in Bacto tryptic soy broth (TSB; BD; catalog no. 211825) under respiratory and nonrespiratory conditions. We observed almost no growth defect in the *S. aureus* ΔG4 mutants under aerobic or nonrespiratory conditions in TSB (including NO stress, anaerobiosis, and metal chelation) (see Fig. S3 in the supplemental material). This suggests that glucose transport is nonessential for growth under nutrient-rich conditions, perhaps because of the presence or uptake of other carbohydrates. In line with this hypothesis, we observed an additive effect of the Δ*glcU* and *ptsH*-H15A mutations under aerobic conditions (see Fig. S3). However, the *ptsH*-H15A Δ*glcU* mutant exhibited drastic growth rate reductions under nonrespiratory conditions, including anaerobiosis, NO·exposure, and metal chelation (see Fig. S3). These data indicate that carbon is not a limiting factor for *S. aureus* in TSB and that uptake of other carbohydrates can compensate for a reduction in *S. aureus* glucose uptake even when high glycolytic flux is required upon respiration inhibition.

### Glucose uptake contributes to *S. aureus* virulence in a murine SSTI model.

**FIG 5:**
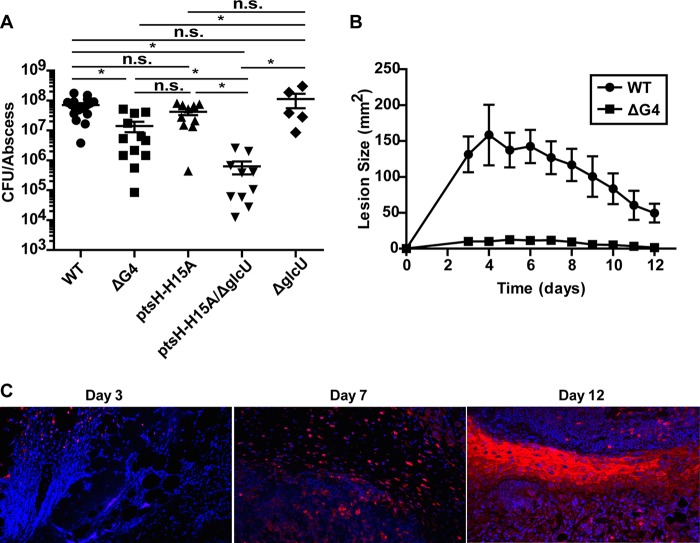
*S. aureus* glucose transporter mutants show attenuated virulence in a murine SSTI model. (A) Abscess burdens on day 5 following the subcutaneous injection of 1 × 10^7^ CFU of *S. aureus* LAC (5 ≤ *n* ≤ 17; error bars show the standard error of the mean). Statistical significance was determined by analysis of variance with multiple comparisons (*, *P* ≤ 0.05). (B) Lesion sizes following the subcutaneous injection of 1 × 10^8^ CFU of *S. aureus* LAC (5 ≤ *n* ≤ 10; error bars show the standard error of the mean). (C) Immunohistochemistry with antibodies against Hypoxyprobe adduct demonstrating the slow progression of abscess tissue from relatively aerobic conditions at early time points toward hypoxic conditions at later time points.

To investigate the relative contributions of glucose and other carbohydrates to *S. aureus* growth or survival during infection, C57BL/6 mice were subcutaneously infected with 1 × 10^7^ CFU of WT or ΔG4, *ptsH*-H15A, *ptsH*-H15A Δ*glcU*, or Δ*glcU* mutant *S. aureus* LAC. At 5 days postinfection, the *S. aureus* ΔG4 mutant was significantly attenuated by the abscess burden (~1 log), while the *ptsH*-H15A mutant, despite losing the functions of three of the four glucose transporters and all other PTS carbohydrate transporters, was not significantly attenuated (Fig. 5A). Combining the Δ*glcU* and *ptsH*-H15A mutations resulted in greater attenuation than *S. aureus* ΔG4 alone (~2-log difference from the WT). This difference was not just due to mutation of the *glcU* allele, as the Δ*glcU* single mutant was also not significantly attenuated in comparison with the WT. Importantly, we observed no reversion of the *ptsH*-H15A mutation over the course of infection.

Interestingly, when we infected mice with a dose (1 × 10^8^ CFU) that results in measurable skin lesions, the *S. aureus* ΔG4 mutant produced almost no abscess formation, unlike the WT (Fig. 5B). However, when grown on blood agar plates, the *S. aureus* ΔG4 mutant exhibited no obvious hemolysis defects (see Fig. S4 in the supplemental material). Altogether, these data suggest that glucose is the primary carbohydrate utilized by *S. aureus* during skin infections and that other carbohydrates contribute minimally to the growth or survival of *S. aureus*. This conclusion is supported by our observation that the elimination of all PTS carbohydrate transport only attenuates *S. aureus* when glucose transport is also limited (Fig. 5A). Factors that necessitate the fermentation of glucose during infection include iron chelation and NO production (13, 15, 16). Additionally, as the abscess progresses, oxygen becomes scarce, further limiting the efficiency of respiration. This becomes apparent with Hypoxyprobe staining as early as day 7 but is overwhelmingly measurable by day 12 (Fig. 5C). Thus, the combination of iron chelation, NO production, and hypoxia within *S. aureus* skin abscesses necessitates fermentative metabolism and the robust import of glucose by the bacterium.

## DISCUSSION

Many variables within host tissue necessitate nonrespiratory growth, including the production of immune radicals, the sequestration of iron, and the inevitable hypoxia that arises at sites of inflammation because of the rapid consumption of oxygen by active immune cells. *S. aureus* has evolved to thrive under all of these stresses provided it has a rich source of carbohydrates, particularly glucose. Under aerobic conditions, respiration contributes directly to PMF, which in turn is used to generate ATP. In the absence of respiration, the only source of ATP is substrate level phosphorylation. Moreover, PMF has to be adequately maintained by consumption of ATP and reversal of the F_1_F_0_-ATPase. Therefore, under nonrespiratory conditions, *S. aureus* requires enhanced glycolytic flux, as demonstrated by a >3-fold increase in glucose consumption (Fig. 3A).

In order to accommodate an elevated level of glycolytic flux, *S. aureus* must efficiently acquire host carbohydrates. Importantly, glucose is the most abundant free carbohydrate in the human body, and elevated host glucose levels are associated with greater *S. aureus* disease (11, 22, 28–30). However, efficient glucose uptake is likely difficult in inflamed tissue spaces, given that infiltrating phagocytes rapidly consume tissue glucose by running a metabolic scheme not unlike Warburg metabolism (i.e., robust glucose oxidation combined with extensive lactate secretion) (31). Infiltrating neutrophils rely very little on the trichloroacetic acid (TCA) cycle or mitochondrial respiration, likely because these energy-efficient pathways are susceptible to the reactive immune radicals produced by these immune cells. By acquiring additional glucose uptake capabilities, as well as a highly active lactate dehydrogenase, *S. aureus* has distinguished itself from other skin-dwelling staphylococci and evolved to “mimic” the metabolic state of the host at sites of inflammation.

It should be noted that glucose is not the only substrate for the *S. aureus*-specific GlcA and GlcC transporters. We found that GlcA and GlcC are solely responsible for the uptake of GlcNAc and ManNAc, respectively, which may be indicative of a role in peptidoglycan homeostasis. However, the selective pressure for these transporters during infection is likely their affinity for glucose. This conclusion is drawn from the fact that the Δ*glcA* Δ*glcC* mutant, which is completely devoid of GlcNAc or ManNAc import, is fully virulent (data not shown). Thus, a role in cell wall homeostasis cannot explain the maintenance of these two genes. Rather, attenuation in the animal model of skin infection requires loss of either all carbohydrate transport (*ptsH*-H15A Δ*glcU*) or specific loss of glucose transport (ΔG4) (Fig. 5A). The fact that the *ptsH*-H15A mutant alone (unable to utilize almost all carbohydrates, with the exception of glucose) is fully virulent implies that all other carbohydrates found within the host environment are incapable of sustaining *S. aureus* in vivo.

In addition to meeting the energy needs of the cell, the effect of glucose on *S. aureus* virulence factor regulation in the context of infection should not be ignored. Specifically, *in vitro* glucose induces the expression of *S. aureus* biofilm-related genes (*cidA* and *icaA*) and modulates the expression of the genes for a master virulence regulator (*agr*/RNAIII), toxins (*hla*, *sec*, and *tst*), and protein A (*spa*) (32–36). This may explain the complete loss of lesion formation in mice infected with the ΔG4 mutant despite only a modest reduction in the viable CFU count (Fig. 5). Although we did not observe a loss of hemolytic activity in *S. aureus* ΔG4 grown *in vitro* on blood agar plates (see Fig. S4 in the supplemental material), this experiment is not quantitative and does not rule out a difference in the kinetics or cumulative levels of toxin production. Similarly, we found that the *S. aureus* ptsH-H15A Δ*glcU* mutant exhibited normal hemolysis but displayed reduced pigment formation. This defect may be explained as follows: (i) slow growth of the mutant in TSB may delay *sigB* activation of the *crtOPQMN* operon, and/or (ii) reduced carbohydrate uptake may limit the intracellular availability of glucose, a required substrate for staphyloxanthin production (37, 38). Regardless, our data indicate that carbohydrate uptake may also contribute to *S. aureus* infection via regulation of virulence factor production.

To contextualize our *in vivo* findings, one must also consider that respiration, iron acquisition, and the TCA cycle have all been shown to contribute to *S. aureus* virulence (39–42). Thus, we cannot accurately state that inflamed tissue spaces are strictly nonrespiratory. However, it is clear from our work that *S. aureus* displays enhanced nonrespiratory growth phenotypes and that glycolysis-based fermentation is equally required for infection. These seemingly paradoxical findings can be reconciled by considering the temporal and spatial aspects of infection. For instance, NO production and oxygen availability are temporally regulated during *S. aureus* abscess development (14). Skin abscess-inducible NO synthase activity is highest 1 to 7 days after *S. aureus* injection and then wanes as the infection clears. However, over time, the abscesses become hypoxic (Fig. 5C). These data suggest that *S. aureus* uses carbohydrate-based fermentative metabolism to overcome instances of high NO exposure encountered early during infection and instances of hypoxia later in infection. Moreover, bacteria within murine renal abscess have been shown to be relatively starved of iron (43). Thus, until bacterial numbers are reached such that efficient hemolysis releases hemoglobin into the tissue, allowing *S. aureus* sufficient iron to respire, *S. aureus* may rely on nonrespiratory metabolism to thrive, necessitating rapid import of glucose.

Regardless of when or why *S. aureus* glycolytic and glucose transporter-deficient mutants are attenuated during infection, the fact that they exhibit any attenuation at all emphasizes the importance of glucose to *S. aureus* disease. In particular, this observation may partially explain the unique susceptibility of uncontrolled diabetics to *S. aureus* infections (30). Diabetes is an important risk factor for *S. aureus* disease, with diabetic individuals exhibiting an increased incidence and severity of *S. aureus* SSTIs, bloodstream infections, and endocarditis (22, 28, 29, 44–46). If the susceptibility of diabetics to *S. aureus* infection is, in fact, augmented by the enhanced propensity of *S. aureus* to acquire and ferment glucose, then the development of novel *S. aureus* glycolysis inhibitors by Kumar et al. may constitute a particularly effective treatment for diabetics with *S. aureus* infections, one that both limits *S. aureus* growth and reduces its destructive capacity during infection (47).

## MATERIALS AND METHODS

### Bacterial strains and medium.

All staphylococci were cultivated in TSB or CDM, wherein the primary carbon source could be modified (48). Individual carbohydrates added to CDM were carbon balanced to 25 mM glucose for all experiments, except the NO growth assay (see explanation below). Casamino Acids were added to the CDM at 0.5%. Chloramphenicol was added to TSB (10 µg/ml) and CDM (2.5 µg/ml) during the growth of plasmid-containing strains. All of the strains utilized in this study are listed in Table S3 in the supplemental material. All mutant strains, except the PTS insertion mutants, were generated via allelic replacement with *Escherichia coli-S. aureus* shuttle vectors pBT2ts, pBTK, pBTE, and pBTS and the new vector pBTT as previously described (49). pBTT was constructed by amplifying the *tetK* allele from *S. aureus* COL plasmid pT181 (tet.3A and tet.3B) and then cloning it into the XmaI site of pBT2ts. The PTS insertion mutants were ordered from the Nebraska Mutant Transposon Library (Network on Antimicrobial Resistance in *Staphylococcus aureus*) and verified by PCR upon arrival (24). For the plasmids and primers used for mutant construction, verification, and complementation, see Table S3. Importantly, all of the mutants used for virulence studies were fully transduced, with the exception of the *ptsH*-H15A and *ptsH-*H15A Δ*glcU* mutants. Since the *ptsH*-H15A mutation is markerless, we constructed and verified three independent *ptsH*-H15A mutants in the LAC background. We then separately transduced the Δ*glcU* mutation into each of these three mutants and verified that each mutant grew identically under aerobic conditions in glucose, Casamino acids, and TSB.

### Growth curves.

*Staphylococcus* cultures were grown overnight in TSB at 37°C with shaking at 250 rpm. For aerobic, metal-restricted, and NO-treated bacterial growth curves, overnight cultures of *S. aureus* were washed twice with phosphate-buffered saline (PBS) and diluted into TSB with or without 2,2-dipyridyl (1 mM) or into CDM with or without carbon to an initial OD at 600 nm (OD_660_) of 0.04. Diluted cultures were then aliquoted into a 96-well plate (200 µl/well) and incubated in a Tecan Infinite M200 microplate reader set to 37°C with 1-mm orbital shaking. Growth was monitored via absorbance at 650 nm every 15 min for 24 h. For NO growth curves, 10 mM NOC-12 (Santa Cruz Biotechnology; catalog no. 202246) and 1 mM DEA NONOate (A. G. Scientific; catalog no. D-1013) were added to the cultures at an OD_650_ of 0.15. To extend the fermentative phase of *S. aureus* NO-resistant growth, an additional, identical, dose of NO donors was added to each well 1.5 h later. To ensure continued substrate availability during such prolonged NO exposure (*S. aureus* utilizes carbon inefficiently during NO-induced fermentation), we used 50 mM glucose for these experiments. For anaerobic growth curves, the overnight cultures were washed twice with PBS and diluted into 5 ml of prewarmed (37°C) TSB or CDM with or without carbon with or without 50 mM potassium nitrate to an OD_660_ of 0.08. Cultures were prepared in duplicate in 16- by 150-mm glass tubes containing 1-mm stir bars. Following dilution, cultures were immediately transferred into a Coy anaerobic chamber and grown at 37°C with stirring. Growth was monitored hourly by reading absorbance at 650 nm.

### Growth rate and lag analysis.

Growth rates were calculated with the formula μ = Δln(*A*_650_) Δtime (hours). The time intervals used for growth rate analysis are experiment specific and thus are provided in the figure legends. Lag time was calculated as the time (hours) until cultures reached an OD_650_ of 0.2.

### Glucose yield calculation.

Glucose yield was measured in milligrams of glucose consumed per milligram (dry weight) of biomass for *S. aureus* COL, our primary laboratory strain. Glucose consumption was monitored by enzymatically (R-Biopharm) determining glucose in 200-µl cultures of CDM plus 25 mM glucose over a 4-h period following NO exposure or during a 4-h period during aerobic or anaerobic growth at mid-exponential phase. Dry-weight biomass was determined by vacuum filtering 100 ml of mid-exponential-phase *S. aureus* COL culture (OD_660_ of 1) in triplicate through a 10-cm Millipore 0.45-µm-pore-size filter. The filter was then baked overnight at 65°C. Weights were averaged, and the weights of baked sterile filters were subtracted to yield an average dry weight of an *S. aureus* cell of ~2.8 × 10^−13^ g. While *S. aureus* LAC (used for animal experiments) exhibits similar elevated glucose consumption under nonrespiratory conditions, the dry-weight biomass of this strain per OD unit was not directly determined, but it is not expect to differ significantly from that of COL.

### Bioinformatic analysis of carbohydrate transporters.

First, we searched the NCBI gene/protein and UniProt database *S. aureus* COL, *S. aureus* LAC, *S. epidermidis* RP62A, *S. haemolyticus* JCSC1435, and *S. saprophyticus* ATCC 15305 genomes with the keywords PTS, sugar transporter, sugar permease, carbohydrate transporter, carbohydrate permease, glucose, fructose, mannose, mannitol, sucrose, galactose, ascorbate, sorbitol, *N*-acetylglucosamine, *N*-acetylmannosamine, ribose, maltose, trehalose, lactose, maltotriose, trisaccharides, disaccharides, and monosaccharides. All of the putative carbohydrate transporters discovered in this manner were then entered as queries in BLASTP searches against all five of the genomes mentioned above.

All of the candidate sugar transporters from this expanded search were then compiled into a list. Next, we performed forward and reciprocal BLASTP searches for each predicted protein on this list against all *Staphylococcus* genomes, as well as the transporter classification database (http://www.tcdb.org). Sequence homology was determined with an E value cutoff of 1e^−50^. Lastly, we used the ortholog predictor provided through xBASE (http://www.xbase.ac.uk/) and visually inspected/compared the genomic context of each gene with MetaCyc (http://metacyc.org/). Homology, as depicted in Table S1 in the supplemental material, required sequence homology (i.e., an E value of <1e^−50^ and a positive reciprocal BLASTP result), a corresponding result from the xBASE ortholog predictor, and a visual confirmation of shared genomic context.

### Real-time qRT-PCR.

*S. aureus* COL was grown in 50 ml of CDM plus 25 mM glucose or Casamino Acids (0.5%) in 250-ml flasks at 37°C with shaking at 250 rpm. At an OD_660_ of 0.5, 25 ml of each culture was added to an equal volume of ice-cold ethanol-acetone (1:1) and frozen at −80°C (aerobic cultures). To assess gene expression during NO exposure, a separate set of cultures (CDM plus glucose) was treated with 5 mM DETA-NONOate (Cayman Chemical; catalog no. 82120) for 1 h, quenched, and then frozen. Lastly, *S. aureus* COL was grown in 50 ml of CDM plus glucose in the anaerobic chamber at 37°C with stirring. At an OD_660_ of 0.5, 25 ml of culture was removed from the chamber in a 50-ml conical tube devoid of oxygen, immediately quenched, and then frozen. RNA was then harvested, and gene expression was analyzed as previously described (49). Transcript levels of selected genes were normalized to *rpo*D transcript levels, which deviated very little across our experimental conditions. For the primers used for quantitative reverse transcriptase PCR (qRT-PCR) analysis, see Table S3 in the supplemental material.

### Radiolabeled-glucose uptake assays.

*S. aureus* COL strains were grown in TSB in 50-ml culture volumes to late exponential phase (OD_660_ of 1 to 1.2). Cells were centrifuged for 10 min at 5,000 × *g* and then immediately resuspended to an OD_660_ of 20 in warm CDM (37°C). At *t*_0_, a mixture of glucose and [^14^C]glucose was added to a 1-ml aliquot of each culture to reach final concentrations of 2 mM glucose and 100 µM [^14^C]glucose. Cells were incubated in a 37°C heat block. At 12 min following glucose addition, 150 µl of culture was removed and immediately diluted into 900 µl of CDM containing 20 mM unlabeled glucose. The diluted cells were pelleted, washed once with 500 µl of CDM (20 mM glucose), and then resuspended in 150 µl of CDM (20 mM glucose). The resuspended cells were added to a scintillation vial containing 4 ml of EcoScint A scintillation fluid (National Diagnostics). To determine the level of radioactivity in each sample, a Beckman LS6500 Multi-Purpose Scintillation Counter was used to measure counts per minute.

### Hemolysis assays.

To detect hemolysis activity, *S. aureus* LAC strains (WT, ΔG4, *ptsH*-H15A [isolates 1 to 3], and *ptsH*-H15A Δ*glc*U [isolates 1 to 3]) were streaked onto blood agar (Remel; tryptic soy agar [TSA] with sheep blood; catalog no. R01200) from freezer stocks and incubated at 37°C for 36 h. Plates were subsequently incubated at 4°C for 12 h and then imaged with a digital microscope.

### Virulence assays.

For virulence assessment, 6- to 8-week-old female C57BL/6 mice from The Jackson Laboratory (Bar Harbor, ME) were anesthetized with tribromoethanol (Avertin, 0.08 mg/kg; Acros Organics; catalog no. 421430100) shaved (on the flank), and injected subcutaneously (on the flank) with 1 × 10^7^ CFU of WT or ΔG4, Δ*glcU*, *ptsH*-H15A, or *ptsH*-H15A Δ*glcU* mutant *S. aureus* LAC in 20 µl of sterile PBS. Importantly, two separate isolates of the *ptsH*-H15A and *ptsH*-H15A Δ*glcU* mutants were used for infection of at least five mice apiece. On day 5, mice were euthanized and the abscesses were removed, homogenized in 500 µl of PBS, and dilution plated on TSA to enumerate CFU. To control for reversion of the *ptsH*-H15A mutation during infection, WT (positive control) and *ptsH*-H15A and *ptsH*-H15A Δ*glc*U mutant abscesses were plated on CDM agar plus sucrose (25 mM), incubated at 37°C for 48 h, and then inspected for colonies.

### Fluorescence immunohistochemistry.

The Hypoxyprobe-1 Omni kit (Hypoxyprobe Inc., Burlington, MA) was used for immunochemical detection of tissue hypoxia. Briefly, mice were injected intraperitoneally with 60 mg/kg pimonidazole HCl 30 min prior to euthanasia. Following euthanasia, infected tissues were fixed in 10% formalin, paraffin embedded, and sectioned (5 µm). Unstained sections were deparaffinized with a series of xylene and ethanol washes, followed by antigen retrieval in boiling 10 mM sodium citrate buffer (pH 6). Tissues were blocked with 10% donkey serum (Jackson ImmunoResearch, West Grove, PA) and subsequently incubated with anti-Hypoxyprobe PAb2627AP (Hypoxyprobe Inc.). The primary antibody was detected by incubation with a biotinylated donkey anti-rabbit antibody, followed by incubation with streptavidin-conjugated Dylight 594 (Jackson ImmunoResearch). Tissues were mounted with ProLong antifade gold containing 4′,6-diamidino-2-phenylindole (Invitrogen, Grand Island, NY) and imaged on an Olympus BX60 fluorescence microscope with iVision software v.4.0.0 (BioVision Technologies, New Minas, Nova Scotia, Canada).

## SUPPLEMENTAL MATERIAL

Figure S1  Residual aerobic growth of the *S. aureus* ΔG4 mutant on glucose is PTS independent. Representative aerobic growth curve of WT and ΔG4, *ptsH*-H15A Δ*glcU*, and *ptsH-*H15A *glk*::Tn mutant *S. aureus* LAC in CDM without added carbon or with 25 mM glucose (Glc; *n =* 1). Residual growth using non-PTS transport is marked by the red bracket. Abs, absorbance. Download Figure S1, TIF file, 1.4 MB

Figure S2  Expression analyses of four *S. aureus* glucose transporters. (A) qRT-PCR analysis of *S. aureus* COL *glcA*, *glcB*, *glcC*, and *glcU* transcript levels at mid-exponential growth phase in CDM with either 25 mM glucose or Casamino Acids (0.5%) as a carbon source. (B) qRT-PCR analysis of *glcA*, *glcB*, *glcC*, and *glcU* transcript levels at mid-exponential growth phase in CDM with 25 mM glucose as a carbon source under aerobic, anaerobic, and NO-stressed conditions. The transcript levels of all genes were normalized to that of *rpoD* (*n =* 3; error bars show the standard error of the mean). Download Figure S2, TIF file, 1.4 MB

Figure S3  Carbohydrate transport is essential for *S. aureus* nonrespiratory growth. Average maximum growth rates of *S. aureus* LAC and isogenic *glcA*, *glcB*, *glcC*, *glcU* (ΔG4) and *ptsH*-H15A Δ*glcU* mutants in TSB under respiratory (aerobic) and nonrespiratory (anaerobic, Fe-chelated [1 mM dipyridyl], and NO-stressed [1 mM DEA-NO–10 mM NOC-12 administered when cultures reached an OD_660_ of 0.15]) conditions (*n =* 3; error bars show the standard error of the mean). Statistical significance was calculated with a Student two-sided *t* test (*, *P* ≤ 0.05; **, *P* ≤ 0.01). Abs, absorbance. Download Figure S3, TIF file, 1.4 MB

Figure S4  Hemolysis and pigmentation of *S. aureus* carbohydrate transporter mutants. Shown are the alpha-hemolysis (A), beta-hemolysis (B), and pigmentation (C) of *S. aureus* LAC carbohydrate transporter mutants. The numbering of the *ptsH*-H15A and *ptsH*-H15A Δ*glcU* mutants indicates independent isolates. Download Figure S4, TIF file, 1.4 MB

Table S1  Conservation of predicted *Staphylococcus* carbohydrate transport proteins. Putative carbohydrate transporters, as determined by NCBI gene/protein and UniProt database searches, were evaluated for transporter classification, genomic context, and homology among *S. aureus* COL, *S. aureus* LAC, *S. epidermidis* RP62A, *S. haemolyticus* JCSC1435, and *S. saprophyticus* ATCC 15305 (see Materials and Methods). *S. aureus* encodes the largest total number of carbohydrate transporters (*n =* 22) and the largest number of unique carbohydrate transporters (*n =* 10). Table S1, DOCX file, 0.2 MB

Table S2  Sugar-specific growth characteristics of *S. aureus* COL ΔG4. Maximum OD_650_, lag time (time to an OD_650_ of 0.2), and maximum growth rate of the *S. aureus* COL ΔG4 mutant on a variety of carbon sources are shown. Table S2, DOCX file, 0.1 MB

Table S3  Strains, plasmids, and primers used in this study. Shown are the origins and/or constructions of the strains and plasmids, as well as all of the oligonucleotide sequences used in this study. Table S3, DOCX file, 0.2 MB
