# Supplementary figures and images for: Expanded Glucose Import Capability Affords Staphylococcus aureus Optimized Glycolytic Flux during Infection
Source: mBio. 2016 Jun 21;7(3):e00296-16. doi: 10.1128/mBio.00296-16 (PMC4916373; doi:10.1128/mBio.00296-16)

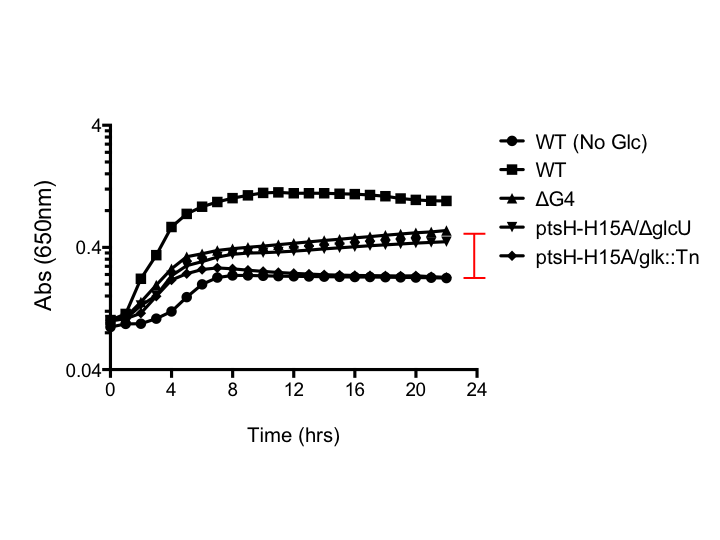

Supplement: Figure S1 — Residual aerobic growth of the S. aureus ΔG4 mutant on glucose is PTS independent. Representative aerobic growth curve of WT and ΔG4, ptsH-H15A ΔglcU, and ptsH-H15A glk::Tn mutant S. aureus LAC in CDM without added carbon or with 25 mM glucose (Glc; n = 1). Residual growth using non-PTS transport is marked by the red bracket. Abs, absorbance. Download [file mbo003162850sf1.tif]

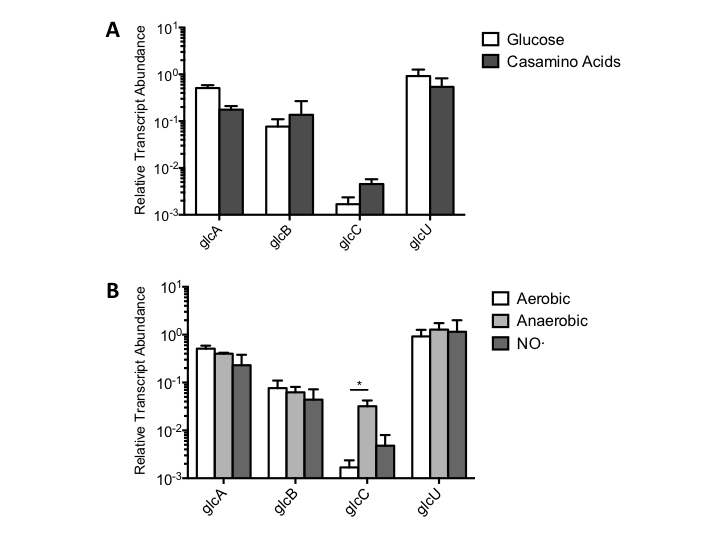

Supplement: Figure S2 — Expression analyses of four S. aureus glucose transporters. (A) qRT-PCR analysis of S. aureus COL glcA, glcB, glcC, and glcU transcript levels at mid-exponential growth phase in CDM with either 25 mM glucose or Casamino Acids (0.5%) as a carbon source. (B) qRT-PCR analysis of glcA, glcB, glcC, and glcU transcript levels at mid-exponential growth phase in CDM with 25 mM glucose as a carbon source under aerobic, anaerobic, and NO-stressed conditions. The transcript levels of all genes were normalized to that of rpoD (n = 3; error bars show the standard error of the mean). Download [file mbo003162850sf2.tif]

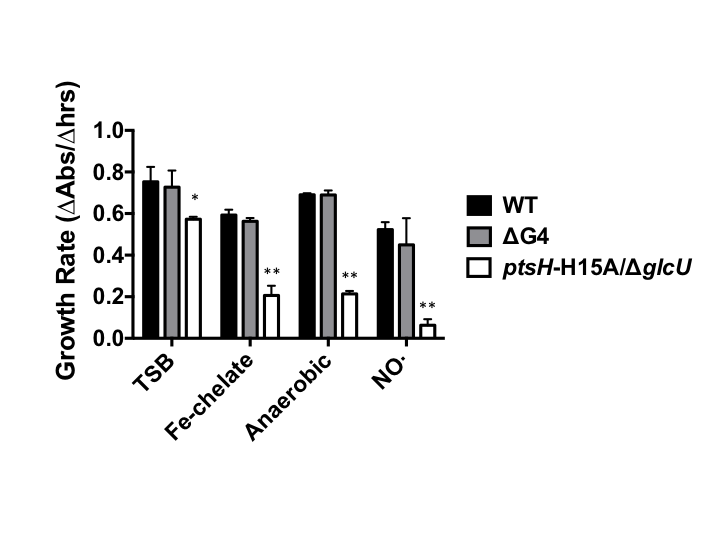

Supplement: Figure S3 — Carbohydrate transport is essential for S. aureus nonrespiratory growth. Average maximum growth rates of S. aureus LAC and isogenic glcA, glcB, glcC, glcU (ΔG4) and ptsH-H15A ΔglcU mutants in TSB under respiratory (aerobic) and nonrespiratory (anaerobic, Fe-chelated [1 mM dipyridyl], and NO-stressed [1 mM DEA-NO–10 mM NOC-12 administered when cultures reached an OD660 of 0.15]) conditions (n = 3; error bars show the standard error of the mean). Statistical significance was calculated with a Student two-sided t test (*, P ≤ 0.05; **, P ≤ 0.01). Abs, absorbance. Download [file mbo003162850sf3.tif]

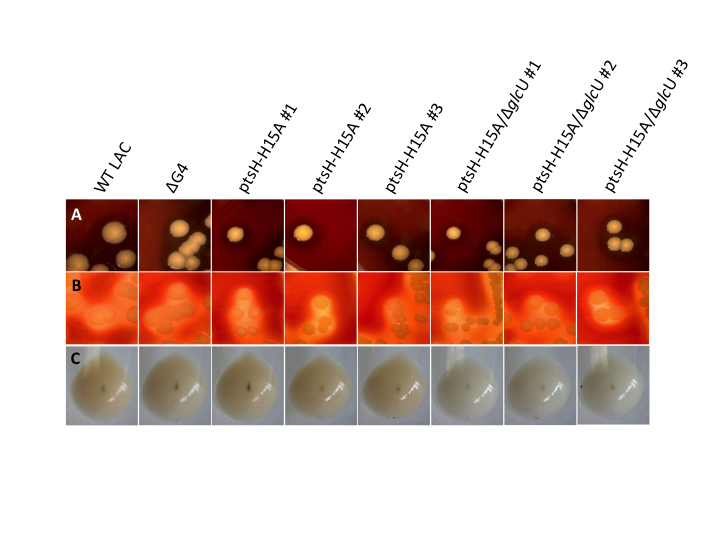

Supplement: Figure S4 — Hemolysis and pigmentation of S. aureus carbohydrate transporter mutants. Shown are the alpha-hemolysis (A), beta-hemolysis (B), and pigmentation (C) of S. aureus LAC carbohydrate transporter mutants. The numbering of the ptsH-H15A and ptsH-H15A ΔglcU mutants indicates independent isolates. Download [file mbo003162850sf4.tif]
