# Supplementary material for: Expanded Glucose Import Capability Affords Staphylococcus aureus Optimized Glycolytic Flux during Infection
Source: mBio. 2016 Jun 21;7(3):e00296-16. doi: 10.1128/mBio.00296-16 (PMC4916373; doi:10.1128/mBio.00296-16)
Supplement: Table S1 — Conservation of predicted Staphylococcus carbohydrate transport proteins. Putative carbohydrate transporters, as determined by NCBI gene/protein and UniProt database searches, were evaluated for transporter classification, genomic context, and homology among S. aureus COL, S. aureus LAC, S. epidermidis RP62A, S. haemolyticus JCSC1435, and S. saprophyticus ATCC 15305 (see Materials and Methods). S. aureus encodes the largest total number of carbohydrate transporters (n = 22) and the largest number of unique carbohydrate transporters (n = 10). [file mbo003162850st1.docx]

**Table S1. Conservation of Predicted Staphylococcus Carbohydrate Transport Proteins.**

| **Organism** | **Locus** | **Gene Name** | **TC #** | **Family**** | **Substrate(s)** | **S. aureus** | | **S. epidermidis** | **S. haemolyticus** | **S. saprophyticus** |
| --- | --- | --- | --- | --- | --- | --- | --- | --- | --- | --- |
|  |  |  |  |  |  | **USA300** | **COL** | **RP62A** | **JCSC1435** | **ATCC 15305** |
| S. aureus (29) | SAUSA300_0191 | *ptsG, glcA* | 4.A.1.1 | PTS-Glc | Glucose | SAUSA300_0191 | SACOL0175 |  |  |  |
|  | SAUSA300_0194 |  | 4.A.1.2 | PTS-Glc |  | SAUSA300_0194 | SACOL0178 | SERP1900 | SH0741 | SSP0594 |
|  | SAUSA300_0208 | *malK* | 3.A.1.1 | CUT1 | Maltose | SAUSA300_0208 | SACOL0192 |  |  |  |
|  | SAUSA300_0216 | *uhpT* | 2.A.1.4.1 | OPA | Hexose-P | SAUSA300_0216 | SACOL0200 |  |  |  |
|  | SAUSA300_0236 | *glcC** | 4.A.1.1 | PTS-Glc |  | SAUSA300_0236 | SACOL0224 |  |  |  |
|  | SAUSA300_0239 |  | 4.A.5.1 | PTS-Gat |  | SAUSA300_0239 | SACOL0229 |  |  |  |
|  | SAUSA300_0240 |  | 4.A.5.1 | PTS-Gat |  | SAUSA300_0240 | SACOL0230 |  |  |  |
|  | SAUSA300_0241 |  | 4.A.5.1 | PTS-Gat |  | SAUSA300_0241 | SACOL0232 |  |  |  |
|  | SAUSA300_0259 |  | 4.A.1.1 | PTS-Glc |  | SAUSA300_0259 | SACOL0250 |  |  |  |
|  | SAUSA300_0264 | *rbsU* | 2.A.7.5.2 | GRP | Ribose | SAUSA300_0264 | SACOL0255 | SERP2102 |  |  |
|  | SAUSA300_0314 | *nanT* | 2.A.21.3 | SSS | Sialic Acid | SAUSA300_0314 | SACOL0311 |  | SH0283 | SSP0376 |
|  | SAUSA300_0330 |  | 4.A.7.1 | PTS-Asc |  | SAUSA300_0330 | SACOL0400 |  |  |  |
|  | SAUSA300_0332 |  | 4.A.7.1 | PTS-Asc |  | SAUSA300_0332 | SACOL0402 |  |  |  |
|  | SAUSA300_0337 | *glpT* | 2.A.1.4.3 | MFS | Glycerol-3-P | SAUSA300_0337 | SACOL0407 | SERP2060 |  |  |
|  | SAUSA300_0448 |  | 4.A.1.2 | PTS-Glu | Fructose | SAUSA300_0448 | SACOL0516 |  | SH2538 | SSP2282 |
|  | SAUSA300_0685 | *fruA* | 4.A.2.1 | PTS-Fru |  | SAUSA300_0685 |  | SERP0359 | SH2194 | SSP2017 |
|  | SAUSA300_1191 | *glpF* | 1.A.8.2 | MIP | Glycerol | SAUSA300_1191 | SACOL1319 | SERP0866 | SH1614 | SSP1461 |
|  | SAUSA300_1315 | *crr* | 4.A.1.1 | PTS-Glc |  | SAUSA300_1315 | SACOL1457 | SERP0998 | SH1484 | SSP1317 |
|  | SAUSA300_1672 |  | 4.A.1.1 | PTS-Glc |  | SAUSA300_1672 | SACOL1775 | SERP1290 | SH1198 | SSP1037 |
|  | SAUSA300_2105 | *mtlA* | 4.A.2.1 | PTS-Fru | Mannitol | SAUSA300_2105 | SACOL2146 |  | SH0235 | SSP0728 |
|  | SAUSA300_2107 | *mtlF* | 4.A.2.1 | PTS-Fru | Mannitol | SAUSA300_2107 | SACOL2148 |  | SH0233 | SSP0726 |
|  | SAUSA300_2150 | *lacE* | 4.A.3.1 | PTS-Lac | Lactose | SAUSA300_2150 | SACOL2181 | SERP1790 | SH0847 |  |
|  | SAUSA300_2151 | *lacF* | 4.A.3.1 | PTS-Lac | Lactose | SAUSA300_2151 | SACOL2182 | SERP1791 | SH0846 |  |
|  | SAUSA300_2210 | *glcU* | 2.A.7.5.1 | GRP | Glucose | SAUSA300_2210 | SACOL2246 | SERP1838 | SH0796 | SSP0657 |
|  | SAUSA300_2270 |  | 4.A.1.1 | PTS-Glc |  | SAUSA300_2270 | SACOL2316 | SERP1909 | SH0732 | SSP0583 |
|  | SAUSA300_2324 |  | 4.A.1.2 | PTS-Glc | Sucrose | SAUSA300_2324 | SACOL2376 | SERP1968 | SH0671 | SSP0512 |
|  | SAUSA300_2449 |  | 2.A.1.14 | ACS | Glucarate | SAUSA300_2449 | SACOL2521 | SERP2069 | SH0566 | SSP0401 |
|  | SAUSA300_2476 | *glcB* | 4.A.1.1 | PTS-Glc | Glucose | SAUSA300_2476 | SACOL2552 | SERP2114 | SH0521 | SSP0336 |
|  | SAUSA300_2576 |  | 4.A.2.1 | PTS-Fru |  | SAUSA300_2576 | SACOL2663 | SERP2260 |  |  |
| S. epidermidis (16) | SERP0025 |  | 2.A.1.1 | SP |  |  |  | SERP0025 |  |  |
|  | SERP0359 | *fruA* | 4.A.2.1 | PTS-Fru |  | SAUSA300_0685 |  | SERP0359 | SH2194 | SSP2017 |
|  | SERP0866 | *glpF* | 1.A.8.2 | MIP | Glycerol | SAUSA300_1191 | SACOL1319 | SERP0866 | SH1614 | SSP1461 |
|  | SERP0998 | *crr* | 4.A.1.1 | PTS-Glc |  | SAUSA300_1315 | SACOL1457 | SERP0998 | SH1484 | SSP1317 |
|  | SERP1290 |  | 4.A.1.1 | PTS-Glc |  | SAUSA300_1672 | SACOL1775 | SERP1290 | SH1198 | SSP1037 |
|  | SERP1790 | *lacE* | 4.A.3.1 | PTS-Lac | Lactose | SAUSA300_2150 | SACOL2181 | SERP1790 | SH0847 |  |
|  | SERP1791 | *lacF* | 4.A.3.1 | PTS-Lac | Lactose | SAUSA300_2151 | SACOL2182 | SERP1791 | SH0846 |  |
|  | SERP1838 | *glcU* | 2.A.7.5.1 | GRP | Glucose | SAUSA300_2210 | SACOL2246 | SERP1838 | SH0796 | SSP0657 |
|  | SERP1900 |  | 4.A.1.2 | PTS-Glc |  | SAUSA300_0194 | SACOL0178 | SERP1900 | SH0741 | SSP0594 |
|  | SERP1909 |  | 4.A.1.1 | PTS-Glc |  | SAUSA300_2270 | SACOL2316 | SERP1909 | SH0732 | SSP0583 |
|  | SERP1968 |  | 4.A.1.2 | PTS-Glc | Sucrose | SAUSA300_2324 | SACOL2376 | SERP1968 | SH0671 | SSP0512 |
|  | SERP2060 | *glpT* | 2.A.1.4.3 | MFS | Glycerol-3-P | SAUSA300_0337 | SACOL0407 | SERP2060 |  |  |
|  | SERP2069 |  | 2.A.1.14 | ACS | Glucarate | SAUSA300_2449 | SACOL2521 | SERP2069 | SH0566 | SSP0401 |
|  | SERP2102 | *rbsU* | 2.A.7.5.2 | GRP | Ribose | SAUSA300_0264 | SACOL0255 | SERP2102 |  |  |
|  | SERP2114 | *glcB* | 4.A.1.1 | PTS-Glc | Glucose | SAUSA300_2476 | SACOL2552 | SERP2114 | SH0521 | SSP0336 |
|  | SERP2260 |  | 4.A.2.1 | PTS-Fru |  | SAUSA300_2576 | SACOL2663 | SERP2260 |  |  |
| S. haemolyticus (20) | SH0071 |  | 2.A.1.1 | SP |  |  |  |  | SH0071 | SSP0532 |
|  | SH0188 |  | 4.A.3.2 | PTS-LAC |  |  |  |  | SH0188 | SSP0240 |
|  | SH0189 |  | 4.A.3.2 | PTS-LAC |  |  |  |  | SH0189 | SSP0238 |
|  | SH0233 | *mtlF* | 4.A.2.1 | PTS-Fru | Mannitol | SAUSA300_2107 | SACOL2148 |  | SH0233 | SSP0726 |
|  | SH0235 | *mtlA* | 4.A.2.1 | PTS-Fru | Mannitol | SAUSA300_2105 | SACOL2146 |  | SH0235 | SSP0728 |
|  | SH0283 | *nanT* | 2.A.21.3 | SSS | Sialic Acid | SAUSA300_0314 | SACOL0311 |  | SH0283 | SSP0376 |
|  | SH0358 |  | 4.A.1.1 | PTS-Glc | Glucose |  |  |  | SH0358 |  |
|  | SH0521 | *glcB* | 4.A.1.1 | PTS-Glc | Glucose | SAUSA300_2476 | SACOL2552 | SERP2114 | SH0521 | SSP0336 |
|  | SH0566 |  | 2.A.1.14 | ACS | Glucarate | SAUSA300_2449 | SACOL2521 | SERP2069 | SH0566 | SSP0401 |
|  | SH0671 |  | 4.A.1.2 | PTS-Glc | Sucrose | SAUSA300_2324 | SACOL2376 | SERP1968 | SH0671 | SSP0512 |
|  | SH0732 |  | 4.A.1.1 | PTS-Glc |  | SAUSA300_2270 | SACOL2316 | SERP1909 | SH0732 | SSP0583 |
|  | SH0741 |  | 4.A.1.2 | PTS-Glc |  | SAUSA300_0194 | SACOL0178 | SERP1900 | SH0741 | SSP0594 |
|  | SH0796 | *glcU* | 2.A.7.5.1 | GRP | Glucose | SAUSA300_2210 | SACOL2246 | SERP1838 | SH0796 | SSP0657 |
|  | SH0846 | *lacF* | 4.A.3.1 | PTS-Lac | Lactose | SAUSA300_2151 | SACOL2182 | SERP1791 | SH0846 |  |
|  | SH0847 | *lacE* | 4.A.3.1 | PTS-Lac | Lactose | SAUSA300_2150 | SACOL2181 | SERP1790 | SH0847 |  |
|  | SH1198 |  | 4.A.1.1 | PTS-Glc |  | SAUSA300_1672 | SACOL1775 | SERP1290 | SH1198 | SSP1037 |
|  | SH1484 | *crr* | 4.A.1.1 | PTS-Glc |  | SAUSA300_1315 | SACOL1457 | SERP0998 | SH1484 | SSP1317 |
|  | SH1614 | *glpF* | 1.A.8.2 | MIP | Glycerol | SAUSA300_1191 | SACOL1319 | SERP0866 | SH1614 | SSP1461 |
|  | SH2194 |  | 4.A.2.1 | PTS-Fru |  | SAUSA300_0685 |  | SERP0359 | SH2194 | SSP2017 |
|  | SH2538 |  | 4.A.1.2 | PTS-Glu | Fructose | SAUSA300_0448 | SACOL0516 |  | SH2538 | SSP2282 |
| S. saprophyticus (21) | SSP0123 |  | 4.A.1.2 | PTS-Glc |  |  |  |  |  | SSP0123 |
|  | SSP0124 |  | 4.A.1.2 | PTS-Glc |  |  |  |  |  | SSP0124 |
|  | SSP0234* |  | 4.A.1.1 | PTS-Glc | Glucose |  |  |  |  | SSP0234* |
|  | SSP0238 |  | 4.A.3.2 | PTS-LAC |  |  |  |  | SH0189 | SSP0238 |
|  | SSP0240 |  | 4.A.3.2 | PTS-LAC |  |  |  |  | SH0188 | SSP0240 |
|  | SSP0241 |  | 4.A.3.2 | PTS-LAC |  |  |  |  |  | SSP0241 |
|  | SSP0336 | *glcB* | 4.A.1.1 | PTS-Glc | Glucose | SAUSA300_2476 | SACOL2552 | SERP2114 | SH0521 | SSP0336 |
|  | SSP0376 | *nanT* | 2.A.21.3 | SSS | Sialic Acid | SAUSA300_0314 | SACOL0311 |  | SH0283 | SSP0376 |
|  | SSP0401 |  | 2.A.1.14 | ACS | Glucarate | SAUSA300_2449 | SACOL2521 | SERP2069 | SH0566 | SSP0401 |
|  | SSP0512 |  | 4.A.1.2 | PTS-Glc | Sucrose | SAUSA300_2324 | SACOL2376 | SERP1968 | SH0671 | SSP0512 |
|  | SSP0532 |  | 2.A.1.1 | SP | Unknown |  |  |  | SH0071 | SSP0532 |
|  | SSP0583 |  | 4.A.1.1 | PTS-Glc |  | SAUSA300_2270 | SACOL2316 | SERP1909 | SH0732 | SSP0583 |
|  | SSP0594 |  | 4.A.1.2 | PTS-Glc |  | SAUSA300_0194 | SACOL0178 | SERP1900 | SH0741 | SSP0594 |
|  | SSP0657 | *glcU* | 2.A.7.5.1 | GRP | Glucose | SAUSA300_2210 | SACOL2246 | SERP1838 | SH0796 | SSP0657 |
|  | SSP0726 | *mtlF* | 4.A.2.1 | PTS-Fru | Mannitol | SAUSA300_2107 | SACOL2148 |  | SH0233 | SSP0726 |
|  | SSP0728 | *mtlA* | 4.A.2.1 | PTS-Fru | Mannitol | SAUSA300_2105 | SACOL2146 |  | SH0235 | SSP0728 |
|  | SSP1037 |  | 4.A.1.1 | PTS-Glc |  | SAUSA300_1672 | SACOL1775 | SERP1290 | SH1198 | SSP1037 |
|  | SSP1317 | *crr* | 4.A.1.1 | PTS-Glc |  | SAUSA300_1315 | SACOL1457 | SERP0998 | SH1484 | SSP1317 |
|  | SSP1461 | *glpF* | 1.A.8.2 | MIP | Glycerol | SAUSA300_1191 | SACOL1319 | SERP0866 | SH1614 | SSP1461 |
|  | SSP2017 | *fruA* | 4.A.2.1 | PTS-Fru |  | SAUSA300_0685 |  | SERP0359 | SH2194 | SSP2017 |
|  | SSP2282 |  | 4.A.1.2 | PTS-Glu | Fructose | SAUSA300_0448 | SACOL0516 |  | SH2538 | SSP2282 |

** glc*C was given this gene name in the current manuscript

** List of Transporter Family Abbreviations:

**ACS**  Anion:Cation Symporter

**CUT1** Carbohydrate Uptake Transporter-1

**GRP** Glucose/Ribose Porter

**MFS** Major Facilitator Superfamily

**MIP** Major Intrinsic Protein

**OPA** Organophosphate:P_i_ Antiporter

**PTS** Phosphotransferase System

**-Glc** Glucose

**-Gat** Galactitol

**-Asc** Ascorbate

**-Fru** Fructose

**-Lac** Lactose

**SP** Sugar Porin

**SSS**  Sodium Solute Symporter
