# Supplementary material for: Expanded Glucose Import Capability Affords Staphylococcus aureus Optimized Glycolytic Flux during Infection
Source: mBio. 2016 Jun 21;7(3):e00296-16. doi: 10.1128/mBio.00296-16 (PMC4916373; doi:10.1128/mBio.00296-16)
Supplement: Table S2 — Sugar-specific growth characteristics of S. aureus COL ΔG4. Maximum OD650, lag time (time to an OD650 of 0.2), and maximum growth rate of the S. aureus COL ΔG4 mutant on a variety of carbon sources are shown. [file mbo003162850st2.docx]

**Table S2. Sugar-specific Growth Characteristics of *S. aureus* COL ∆G4.**

| **Carbon** | **Max Abs (650 nm)** | | **Lag (hrs)** | | **Max µ** | |
| --- | --- | --- | --- | --- | --- | --- |
|  | **WT** | **∆G4** | **WT** | **∆G4** | **WT** | **∆G4** |
| - | 0.24 | 0.25 | N/A | N/A | 0.28 | 0.32 |
| Glucose | 1.07 | 0.63* | 9.05 | 12.61* | 0.72 | 0.44* |
| Glucose-6P | 1.10 | 1.08 | 8.36 | 8.75 | 0.73 | 0.73 |
| Fructose | 0.52 | 0.46* | 14.64 | 17.26* | 0.34 | 0.42 |
| Fructose-6P | 1.08 | 1.07 | 10.57 | 11.00 | 0.65 | 0.64 |
| Mannose | 0.96 | 1.01 | 13.66 | 12.78 | 0.30 | 0.43 |
| Mannitol | 1.03 | 1.06 | 9.78 | 10.27 | 0.62 | 0.61 |
| Galactose | 0.51 | 0.42* | 16.88 | 18.24* | 0.29 | 0.26* |
| Maltose | 1.14 | 1.24 | 9.05 | 9.56 | 0.65 | 0.66 |
| Sucrose | 1.21 | 1.25 | 10.05 | 10.63 | 0.57 | 0.60 |
| Lactose | 1.01 | 1.04 | 12.63 | 12.99 | 0.54 | 0.62 |
| Turanose | 0.26 | 0.24 | N/A | N/A | 0.28 | 0.22 |
| Tehalose | 1.18 | 1.11 | 13.20 | 13.99 | 0.44 | 0.50 |
| Casamino Acids | 0.97 | 0.98 | 9.62 | 9.62 | 0.67 | 0.69 |

* Exhibited significantly poorer utilization than WT, Student’s t-test, two-tailed, p ≤ 0.05.
