# Supplementary material for: Expanded Glucose Import Capability Affords Staphylococcus aureus Optimized Glycolytic Flux during Infection
Source: mBio. 2016 Jun 21;7(3):e00296-16. doi: 10.1128/mBio.00296-16 (PMC4916373; doi:10.1128/mBio.00296-16)
Supplement: Table S3 — Strains, plasmids, and primers used in this study. Shown are the origins and/or constructions of the strains and plasmids, as well as all of the oligonucleotide sequences used in this study. [file mbo003162850st3.docx]

**Table S3. Strains, Plasmids and Primers used in this study**

| **Strain** | **Genotype** | **Reference/Source** |
| --- | --- | --- |
| *S. aureus* COL | Methicillin resistant *S. aureus* clinical isolate, widely used as a laboratory strain | W. Shafer |
| AR1315 | WT *S. aureus* COL + pOS1-plgt plasmid | This Study |
| AR1268 | *S. aureus* COL ∆*glc*A::Kn^R^ | This Study |
| AR1267 | *S. aureus* COL ∆*glc*B::Er^R^ | This Study |
| AR1269 | *S. aureus* COL ∆*glc*C::Sp^R^ | This Study |
| AR1270 | *S. aureus* COL ∆*glc*U::Er^R^ | This Study |
| AR1275 | *S. aureus* COL ∆*glc*A::Kn^R^ (Φ80), ∆*glc*B::Er^R^ | This Study |
| AR1277 | *S. aureus* COL ∆*glc*A::Kn^R^ (Φ80), ∆*glc*C::Sp^R^ | This Study |
| AR1278 | *S. aureus* COL ∆*glc*A::Kn^R^, ∆*glc*U::Er^R^ (Φ80) | This Study |
| AR1276 | *S. aureus* COL ∆*glc*B::Er^R^ (Φ80), ∆*glc*C::Sp^R^ | This Study |
| AR1279 | *S. aureus* COL ∆*glc*C::Sp^R^, ∆*glc*U::Er^R^ (Φ80) | This Study |
| AR1280 | *S. aureus* COL ∆*glc*A::Kn^R^ (Φ80), ∆*glc*B::Er^R^ (Φ80), ∆*glc*C::Sp^R^ | This Study |
| AR1281 | *S. aureus* COL ∆*glc*A::Kn^R^ (Φ80), ∆*glc*B::Er^R^, ∆*glc*U::Er^R^ | This Study |
| AR1283 | *S. aureus* COL ∆*glc*A::Kn^R^ (Φ80), ∆*glc*C::Sp^R^, ∆*glc*U::Er^R^ (Φ80) | This Study |
| AR1282 | *S. aureus* COL ∆*glc*B::Er^R^ (Φ80), ∆*glc*C::Sp^R^, ∆*glc*U::Er^R^ | This Study |
| AR1284 | *S. aureus* COL ∆G4: ∆*glc*A::Kn^R^ (Φ80), ∆*glc*B::Er^R^ (Φ80), ∆*glc*C::Sp^R^, ∆*glc*U::Er^R^) | This Study |
| AR1501 | *S. aureus* ∆G4 + pMG027 | This Study |
| AR1502 | *S. aureus* ∆G4 + pMG028 | This Study |
| AR1503 | *S. aureus* ∆G4 + pMG029 | This Study |
| AR1504 | *S. aureus* ∆G4 + pMG030 | This Study |
| AR1316 | *S. aureus* COL ptsH-H15A | This Study |
| AR1328 | *S. aureus* COL COL0175::Tn (Φ11 NE172) | This Study |
| AR1329 | *S. aureus* COL COL0178::Tn (Φ11 NE112) | This Study |
| AR1330 | *S. aureus* COL COL0224::Tn (Φ11 NE450) | This Study |
| AR1331 | *S. aureus* COL COL0229::Tn (Φ11 NE1421) | This Study |
| AR1332 | *S. aureus* COL COL0230::Tn (Φ11 NE1620) | This Study |
| AR1333 | *S. aureus* COL COL0250::Tn (Φ11 NE1584) | This Study |
| AR1334 | *S. aureus* COL COL0516::Tn (Φ11 NE1490) | This Study |
| AR1335 | *S. aureus* COL COL1457::Tn (Φ11 NE1944) | This Study |
| AR1336 | *S. aureus* COL COL1775::Tn (Φ11 NE1457) | This Study |
| AR1337 | *S. aureus* COL COL1917::Tn (Φ11 NE634) | This Study |
| AR1338 | *S. aureus* COL COL2146::Tn (Φ11 NE929) | This Study |
| AR1339 | *S. aureus* COL COL2148::Tn (Φ11 NE1737) | This Study |
| AR1340 | *S. aureus* COL COL2182::Tn (Φ11 NE61) | This Study |
| AR1341 | *S. aureus* COL COL2316::Tn (Φ11 NE648) | This Study |
| AR1342 | *S. aureus* COL COL2376::Tn (Φ11 NE767) | This Study |
| AR1343 | *S. aureus* COL COL2552::Tn (Φ11 NE39) | This Study |
| *S. aureus* LAC | Methicillin resistant *S. aureus* clinical isolate, widely used as a laboratory strain | This Study |
| AR1432 | *S. aureus* LAC ∆*glc*U::Tc^R^ (Φ11) | This Study |
| AR1297 | *S. aureus* LAC ∆G4: ∆*glc*A::Kn^R^ (Φ11), ∆*glc*B::Er^R^ (Φ11), ∆*glc*C::Sp^R^ (Φ11), ∆*glc*U::Tc^R^ (Φ11) | This Study |
| AR1318 | *S. aureus* LAC ptsH-H15A-1 | This Study |
| AR1319 | *S. aureus* LAC ptsH-H15A-2 | This Study |
| AR1320 | *S. aureus* LAC ptsH-H15A-3 | This Study |
| AR1321 | *S. aureus* LAC ptsH-H15A-1, *glk*::Tn (Φ11 NE759) | This Study |
| AR1323 | *S. aureus* LAC ptsH-H15A-1, ∆*glc*U::Tc^R^ (Φ11) | This Study |
| AR1325 | *S. aureus* LAC ptsH-H15A-2, ∆*glc*U::Tc^R^ (Φ11) | This Study |
| AR1326 | *S. aureus* LAC ptsH-H15A-3, ∆*glc*U::Tc^R^ (Φ11) | This Study |
| *S. aureus* JE2 | Methicillin resistant *S. aureus* laboratory strain | (1) |
| NE39 | SAUSA300_2476::Tn | (1) |
| NE61 | SAUSA300_2150::Tn | (1) |
| NE112 | SAUSA300_0194::Tn | (1) |
| NE172 | SAUSA300_0191::Tn | (1) |
| NE450 | SAUSA300_0236::Tn | (1) |
| NE634 | SAUSA300_1809::Tn | (1) |
| NE648 | SAUSA300_2270::Tn | (1) |
| NE767 | SAUSA300_2324::Tn | (1) |
| NE929 | SAUSA300_2105::Tn | (1) |
| NE992 | SAUSA300_0241::Tn | (1) |
| NE1282 | SAUSA300_0332::Tn | (1) |
| NE1290 | SAUSA300_2576::Tn | (1) |
| NE1421 | SAUSA300_0239::Tn | (1) |
| NE1457 | SAUSA300_1672::Tn | (1) |
| NE1490 | SAUSA300_0448::Tn | (1) |
| NE1584 | SAUSA300_0259::Tn | (1) |
| NE1620 | SAUSA300_0240::Tn | (1) |
| NE1737 | SAUSA300_2107::Tn | (1) |
| NE1927 | SAUSA300_2151::Tn | (1) |
| NE1944 | SAUSA300_1315::Tn | (1) |
| *S. epidermidis* RP62A | Methicillin resistant *S. epidermidis* laboratory strain, ATCC 35984 | ATCC |
| *S. epidermidis* ATCC 12228 | *S. epidermidis* laboratory strain | ATCC |
| T6531 | *S. epidermidis* clinical isolate | (2) |
| W6903 | *S. epidermidis* clinical isolate | (2) |
| W6293 | *S. epidermidis* clinical isolate | (2) |
| *S. haemolyticus* ATCC 29970 | *S. haemolyticus* laboratory strain | ATCC |
| *S. saprophyticus* ATCC 15305 | *S. saprophyticus* laboratory strain | ATCC |
| CIL3 | *S. saprophyticus* clinical isolate | (2) |
| CIL4 | *S. saprophyticus* clinical isolate | (2) |

| **Plasmid** | **Description** | **Reference** |
| --- | --- | --- |
| pBT2ts | *E. coli*/*S. aureus* shuttle vector | (3) |
| pBTE | 1.2 kb *erm*B allele cloned into *Sma*I of pBT2ts | (4) |
| pBTK | 1.4 kb *aph*-A3 allele clones into *Sma*I of pBT2ts | (4) |
| pBTS | 1.3 kb *aad*9 allele cloned into *Sma*I of pBT2ts | (4) |
| pBTT* | 1.7 kb *tet*K allele cloned into *Xma*I of pBT2ts | This study |
| pOS1-plgt | *S. aureus* complementation vector driven by the *lgt* promoter | (5) |
| pMG027 | *glc*B cloned into the NdeI site of pOS1-plgt via Gibson assembly | This study |
| pMG028 | *glc*A cloned into the NdeI site of pOS1-plgt via Gibson assembly | This study |
| pMG029 | *glc*C cloned into the NdeI site of pOS1-plgt via Gibson assembly | This study |
| pMG030 | *glc*U cloned into the NdeI site of pOS1-plgt via Gibson assembly | This study |
| pNV62 | 5' and 3' regions of *glc*B cloned into EcoRI and BamHI sites of pBTE to yield ∆*glc*B::Er^R^ | This study |
| pNV63 | 5' and 3' regions of *glc*A cloned into EcoRI and BamHI sites of pBTK to yield ∆*glc*A::Kn^R^ | This study |
| pNV64 | 5' and 3' regions of *glc*C cloned into EcoRI and BamHI sites of pBTS to yield ∆*glc*C::Sp^R^ | This study |
| pNV65 | 5' and 3' regions of *glc*U cloned into EcoRI and BamHI sites of pBTE to yield ∆*glc*U::Er^R^ | This study |
| pNV66 | 5' and 3' regions of *glc*U cloned into EcoRI and BamHI sites of pBTT* to yield ∆*glc*U::Tc^R^ | This study |
| pNV68 | ptsH (43C>A, 44A>C, 45C>A) was amplified by overlap extension PCR and cloned into EcoRI site of pBT2ts | This study |

130

| **Primers** | **Sequence** | **Use** |
| --- | --- | --- |
| tet.1A | CACTACCCGGGCGCCAGTCGATTTAACGGAC | Construction of pBTT |
| tet.1B | CACTACCCGGGGTTAATACGTGTGCTCTGCGAGG | Construction of pBTT |
| glcA-5.1A | tgcttgaattcGATAATCCCGTATCTGGTCTTGG | Construction of pNV63 |
| glcA-5.1B | tgcttgaattcTTACCAATACGTTGCAATTGACCG | Construction of pNV63 |
| glcA-3.1A | taggtggatccCGCCTATTATCGTGACACAAGG | Construction of pNV63 |
| glcA-3.1B | taggtggatccATAGGCTGCTTCGCTTGATGC | Construction of pNV63 |
| glcB-5.1A | tgcttgaattcGCAGATGTAGGTACAGCAACAG | Construction of pNV62 |
| glcB-5.1B | tgcttgaattcTACCAGCTGCTGGTAAAATCGC | Construction of pNV62 |
| glcB-3.1A | taggtggatccCAAGATGCTGATCCAGGTAAGC | Construction of pNV62 |
| glcB-3.1B | taggtggatccATGACACACGCTACACTGATCG | Construction of pNV62 |
| glcC-5.1A | taggtggatccCAAAGCGTTCAATCACGTGATCG | Construction of pNV64 |
| glcC-5.1B | taggtggatccGACTTGCCGAACTGCTGTGC | Construction of pNV64 |
| glcC-3.1A | tgcttgaattcCAGCAAGGCACTGGTGTGC | Construction of pNV64 |
| glcC-3.1B | tgcttgaattcCGTATGCAACATGGAAATTCTCTGG | Construction of pNV64 |
| glcU-5.1A | tagctgaattcCGATGCGATTGAATCACCTGG | Construction of pNV65 and pNV66 |
| glcU-5.1B | tagctgaattcCACTTCCCCAGAATAAAGCAGG | Construction of pNV65 and pNV66 |
| glcU-3.1A | tagctggatccAAGATCGTCGTCAGATGACGG | Construction of pNV65 and pNV66 |
| glcU-3.1B | tagctggatccTTATGGCTGATATAGGTGGTGC | Construction of pNV65 and pNV66 |
| ptsH-H15A-5.1A | ctaggatccGATCGTGATTGATCCACCTAGC | Construction of pNV68 |
| ptsH-H15A-5.1B | GGTCTAGC*TGC*AATACCAGTC | Construction of pNV68 |
| ptsH-H15A-3.1A | GACTGGTATT*GCA*GCTAGACC | Construction of pNV68 |
| ptsH-H15A-3.1B | ctaggatccAAGCTCTGCGTGAACACCATC | Construction of pNV68 |
| glcB-lgt.1a | atacaattgaggtgaacatATGTTTAAGAAATTGTTTGGAC | Construction of pMG027 |
| glcB-lgt.1b | tttggatcctcgagcatatgTTATTTGACTGTCATAATTAGCTTAC | Construction of pMG027 |
| glcA-lgt.1a | atacaattgaggtgaacatATGAGGAAGAAACTTTTCG | Construction of pMG028 |
| glcA-lgt.1b | tttggatcctcgagcatatgTTATTTAGCTTCAAATAATTGATC | Construction of pMG028 |
| glcC-lgt.1a | atacaattgaggtgaacatATGAAATCTTTATTTGAAAAAGC | Construction of pMG029 |
| glcA-lgt.1b | tttggatcctcgagcatatgTTAATCCCCGAGCAATTC | Construction of pMG029 |
| glcU-lgt.1a | atacaattgaggtgaacatATGCAATTTCTTGATTTCTTAATC | Construction of pMG030 |
| glcU-lgt.1b | tttggatcctcgagcatatgCTATTTCAAATTACCTAGAATTATAGC | Construction of pMG030 |
| rpoD_RT.1A | AACTGAATCCAAGTGATCTTAGTG | qRT-PCR |
| rpoD_RT.1B | TCATCACCTTGTTCAATACGTTTG | qRT-PCR |
| glcA-RT.1A | CTGTTGCGATTTTACCAGCAGC | qRT-PCR |
| glcA-RT.1B | GATACCACCAGCACCTGTC | qRT-PCR |
| glcB.1A | TTGCGATTTTACCAGCAGCTGG | qRT-PCR |
| glcB-RT.1B | ACAACTTGTCCTGCTGCTTCC | qRT-PCR |
| glcC-RT.2A | GGTCATCTTTGCAATTGGTGTCG | qRT-PCR |
| glcC-RT.2B | ATCTTTTGCCAATGTGCCCGTG | qRT-PCR |
| glcU-RT.1A | TTCGTCGGCGGTGGACC | qRT-PCR |
| glcU-RT.1B | CAAACGCCCATAATGCACCAG | qRT-PCR |
| NE39-R | ATGTGCTGGTTCTTTCGCTGC | Confirmation of Tn insertion |
| NE61-F | ATACAGTGTAACGATGATGCATGG | Confirmation of Tn insertion |
| NE61-R | AAGATTTCGCTGTCGTTCCTGC | Confirmation of Tn insertion |
| NE112-F | CTGAGCATGATGTGAAAGTTGCG | Confirmation of Tn insertion |
| NE112-R | ATTGACTGTACCAGGTCCAACC | Confirmation of Tn insertion |
| NE172-F | GGTGGCGTACAAACTGTCGC | Confirmation of Tn insertion |
| NE172-R | GAAACGCTTACCAGCGAAGAAACC | Confirmation of Tn insertion |
| NE450-F | CCAAACACCGTTAAAGCATACCC | Confirmation of Tn insertion |
| NE450-R | CGGTCATAATACCTGTGATAATCC | Confirmation of Tn insertion |
| NE634-F | CTATTGGTGCTGGTGTTGTTATTGC | Confirmation of Tn insertion |
| NE634-R | ACCCCCACTAACGATAGGTAC | Confirmation of Tn insertion |
| NE648-F | ACAATGGCCACATACGTTTGGC | Confirmation of Tn insertion |
| NE648-R | CATACACCGATATAGCCTGAAGC | Confirmation of Tn insertion |
| NE767-F | CGGAAGTCAAAGCACAATCTGC | Confirmation of Tn insertion |
| NE767-R | CGTATGCGCTCATTAGCGATGG | Confirmation of Tn insertion |
| NE929-F | GAGGTGTAAACATGTCACAAACTG | Confirmation of Tn insertion |
| NE929-R | GTGGACCCATAATCATTGCACC | Confirmation of Tn insertion |
| NE992-F | CCAGCAGCACAAGCGATGG | Confirmation of Tn insertion |
| NE992-R | CATCGGTGCTGTCCAATCTGC | Confirmation of Tn insertion |
| NE1282-F | CATTGGGACAGTTAAGCGAATGC | Confirmation of Tn insertion |
| NE1282-R | CATTGTTCGGTCTTGCATGTGC | Confirmation of Tn insertion |
| NE1290-F | ACTTGGATGATGATTCAGTGAACC | Confirmation of Tn insertion |
| NE1290-R | ATGACACCGTCAGCTTCTTCG | Confirmation of Tn insertion |
| NE1421-F | AGGGAAAATGGATGGGACAGC | Confirmation of Tn insertion |
| NE1421-R | TTCTGTCTGTTGATCAAGTGTTCC | Confirmation of Tn insertion |
| NE1457-F | AACGTCTAGTTCCAATTATGACTGC | Confirmation of Tn insertion |
| NE1457-R | GTATGATACATTGCTAACGCAGCC | Confirmation of Tn insertion |
| NE1490-F | ATTAATGTGATTGCGAGTACGGC | Confirmation of Tn insertion |
| NE1490-R | AAGCGCTACGGGTCCAACG | Confirmation of Tn insertion |
| NE1584-F | ATGCAACGTATGATACGAAGTGC | Confirmation of Tn insertion |
| NE1584-R | GATTGCTAAGCCTTCACCTACC | Confirmation of Tn insertion |
| NE1620-F | AAATTGGAGCAGATTCTACGAACG | Confirmation of Tn insertion |
| NE1620-R | GACCCACGTTACATATCAATTCCG | Confirmation of Tn insertion |
| NE1737-F | TCAAGCAATGAAAGATCGTGAGC | Confirmation of Tn insertion |
| NE1737-R | CCAGCACCAAAGTGAACTGC | Confirmation of Tn insertion |
| NE1927-F | TAACAGCAGCTCAAGCTGGAG | Confirmation of Tn insertion |
| NE1927-R | AATAAATCCATCACGGATGGCTC | Confirmation of Tn insertion |
| NE1944-F | CACAGTTCAATTAGACGGTGAAGG | Confirmation of Tn insertion |
| NE1944-R | GTCAAATATCTCTTCTGCTAGACG | Confirmation of Tn insertion |

1. **Fey PD**, **Endres JL**, **Yajjala VK**, **Widhelm TJ**, **Boissy RJ**, **Bose JL**, **Bayles KW**. 2012. A Genetic Resource for Rapid and Comprehensive Phenotype Screening of Nonessential Staphylococcus aureus Genes. mBio **4**:e00537–12–e00537–12.

2. **Richardson AR**, **Libby SJ**, **Fang FC**. 2008. A Nitric Oxide-Inducible Lactate Dehydrogenase Enables Staphylococcus aureus to Resist Innate Immunity. Science **319**:1672–1676.

3. **Brückner R**. 1997. Gene replacement in Staphylococcus carnosus and Staphylococcus xylosus. FEMS Microbiol Lett **151**:1–8.

4. **Fuller JR**, **Vitko NP**, **Perkowski EF**, **Scott E**, **Khatri D**, **Spontak JS**, **Thurlow LR**, **Richardson AR**. 2011. Identification of a lactate-quinone oxidoreductase in Staphylococcus aureus that is essential for virulence. Front Cell Infect Microbiol **1**:19.

5. **Bubeck Wardenburg J**, **Williams WA**, **Missiakas D**. 2006. Host defenses against Staphylococcus aureus infection require recognition of bacterial lipoproteins. Proc Natl Acad Sci USA **103**:13831–13836.
